# Sarah Blandy, Ann Dupuis and Jennifer Dixon (eds): Multi-owned housing: law, power and practice: Ashgate, Farnham, Surrey, UK, 2010, 241 pp, ISBN 9780754675662

**DOI:** 10.1007/s10901-012-9286-5

**Published:** 2012-04-03

**Authors:** Frits Meijer

**Affiliations:** OTB Research Institute of the Built Environment, Delft University of Technology, Delft, The Netherlands

*Multi*-*Owned Housing: Law, Power and Practice* explores the issues of private governance and management with respect to multi-owned housing. The foundations of the book were laid in 2005 with a conference paper in which the editors analysed multi-owned developments in England and New Zealand. The aim of this book is to provide a thorough conceptual basis on which the governance and management of housing with multiple owners can be examined across different jurisdictions in various countries.

The choice of countries was partly based on practical considerations. The editors opted for countries with a common law regime and English language tradition. These rather arbitrary criteria may explain a certain imbalance as regards the book’s contents: five of the twelve country chapters deal with New Zealand and Australia. The other seven refer to England & Wales, Scotland, the United States, Israel, Singapore, Hong Kong and China. Besides its international perspective, interdisciplinarity is a key aspect of this book. As the backgrounds of the contributors suggest, the subject is relevant to scholars from a range of scientific disciplines.

The introductory chapter presents a short and pragmatic conceptual framework for studying multi-owned housing. Its key concepts are rights and obligations and its focus is on the way these are allocated to the stakeholders during the life span of a complex. This conceptual framework is not applied consistently throughout the book, however, which makes it difficult to draw comparisons between the countries studied. Nonetheless, the book is informative and the case studies provide good insight in the issues the various countries encounter with regard to multi-owned housing.

The book kicks off with England & Wales (Blandy). Following the conceptual framework, Blandy explores a wide range of statutory rules that apply to multi-owned residential projects and their owners’ experiences. Irrespective of the legal framework, individual owners find themselves in a far weaker position than the developers involved. Although this power imbalance has been reduced slightly by legislative reforms, further improvements seem necessary. Besides statutory changes, Blandy advocates initiatives that should lead to better-informed purchasers. A similar conclusion is drawn for Scotland (by Robertson). Although major reforms have recently changed the property system, the interests of developers and managers are still better safeguarded than those of the occupants. Robertson also goes into the negative effects that statutory requirements have on housing conditions. The Scottish legal arrangements are inadequate for proper governance, and this directly contributes to the very poor quality and largely unaccountable management services offered to owners of multi-owned complexes. The situation in the US (McKenzie) seems to be just the opposite. The relations between homeowner associations (CID’s) and the occupants are viewed as a private matter. Based on case studies in three US states, McKenzie concludes that these private relationships require more public oversight and a stronger regulatory framework.

The chapter on Israel (Alterman) zooms in on the quality of multi-owned housing. She elaborates on the effectiveness of ‘simple’ (in Israel) versus ‘enhanced’ (in the US state of Florida) condominium laws to ensure long-term financing for long-term maintenance. The overall conclusion is not clear-cut: neither of the two types is adequate. Although enhanced laws provide a far better legal framework in stable economic situations, they are less flexible when facing an economic crisis. The fees the owners have to pay can overburden them. In her main recommendation, Alterman states the obvious: do not build tower condominiums! If policymakers nevertheless want to go that direction, an enhanced law should regulate their maintenance and a public body should monitor the financial means and range of the maintenance fund.

The following chapters analyse residential multi-owned housing in Asia. Wang elaborates on the recently instituted but inadequate statutory framework for the management of new multi-owned housing in China. The power relationships between the stakeholders are hugely unbalanced. Again, owners lack power vis-à-vis developers and property management firms. In Hong Kong (Yip) a similar unbalanced legal framework fails to properly empower the occupants. In Singapore (Christudason) the problem lies in the power imbalance of minority residential owners versus majority commercial owners. The current statutory framework offers the minority owners inadequate legal protection in, for instance, decisions to sell mixed-use buildings.

The final five chapters explore a variety of issues in multi-owned housing in Australia and New Zealand. The chapters on Australia deal with residential satisfaction in Sydney (Bounds), long-term management (Sherry) and multi-titled tourism accommodations in Queensland (Cassidy et al.). The common denominator of these contributions is that they—again—highlight the unbalanced power situation during the life course of multi-owned projects. The two chapters on New Zealand elaborate on new forms of private governance for multi-owned housing (Dupuis & Dixon) and describe models for private governance of sustainable design features (Dixon & Van Roon).

Despite differences in context and jurisdiction, the practical experiences in the countries studied are remarkably similar. The regulatory systems for multiple ownership give developers far more power than owners and occupants. Some serious problems need to be straightened out to allow positive changes in the future. The problems are not confined to the current statutory mechanisms but include the improvement of consumer protection, the development of ways to deal with mutual disagreements and the provision of unambiguous and comprehensible information for prospective purchasers (and other parties involved). An essential topic for the near future is the quality of multi-owned complexes. Although quality aspects are only touched upon in this book, the concluding chapter mentions maintenance as a vital component of any research agenda. The topic is explicitly related to the role of local authorities and the roles and obligations of individual owners. This approach should be broadened. An important task for the near future is to safeguard the physical and energetic quality of the multi-owned housing sector. The sector has grown rapidly over the past few decades and is expected to keep growing. The research focus must not only be on public law and the role of (public) authorities. It should also include the instruments that could be provided by private parties and the roles they could play. Besides physical quality, the future prospects of this sector also deserve attention. In the Netherlands, for instance, a considerable portion of the multi-owned housing sector consists of small dwellings in unattractive neighbourhoods. It is important to prevent these parts of the housing market from spiralling downward. Learning from experiences in other countries and sharing findings and recommendations from national research projects could bring us all further. This book provides a sound and valuable foundation on which the research work of scholars from different disciplines can be based.

